# Cancer incidence in people with HIV in Italy: Comparison of the ICONA COHORT with general population data

**DOI:** 10.1002/ijc.35493

**Published:** 2025-05-29

**Authors:** Pierluca Piselli, Alessandro Tavelli, Claudia Cimaglia, Camilla Muccini, Alessandra Bandera, Giulia C. Marchetti, Carlo Torti, Valentina Mazzotta, Luca Pipitò, Alessandro Caioli, Enrico Girardi, Andrea Antinori, Diego Serraino, Antonella d'Arminio Monforte, Antonella Cingolani, Icona Foundation Study Group, A d'Arminio Monforte, A Antinori, S Antinori, A Castagna, R Cauda, G Di Perri, E Girardi, R Iardino, A Lazzarin, GC Marchetti, C Mussini, E Quiros‐Roldan, L Sarmati, B Suligoi, F von Schloesser, P Viale, A d'Arminio Monforte, A Antinori, A Castagna, F Ceccherini‐Silberstein, A Cingolani, A Cozzi‐Lepri, A Di Biagio, E Girardi, A Gori, S Lo Caputo, G Marchetti, F Maggiolo, C Mussini, M Puoti, CF Perno, C Torti, A Antinori, A Bandera, S Bonora, A Calcagno, D Canetti, A Castagna, F Ceccherini‐Silberstein, A Cervo, A Cingolani, P Cinque, A Cozzi‐Lepri, A d'Arminio Monforte, A Di Biagio, R Gagliardini, A Giacomelli, E Girardi, N Gianotti, A Gori, G Guaraldi, S Lanini, G Lapadula, M Lichtner, A Lai, S Lo Caputo, G Madeddu, F Maggiolo, V Malagnino, G Marchetti, A Mondi, V Mazzotta, C Mussini, S Nozza, CF Perno, S Piconi, C Pinnetti, M Puoti, E Quiros Roldan, R Rossotti, S Rusconi, MM Santoro, A Saracino, L Sarmati, V Spagnuolo, N Squillace, V Svicher, L Taramasso, C Torti, A Vergori, A Cozzi‐Lepri, S De Benedittis, I Fanti, M Giotta, C Marelli, A Rodano, A Tavelli, M Cernuschi, L Cosmaro, A Perziano, V Calvino, D Russo, M Farinella, N Policek, VL Del Negro, M Augello, S Carrara, S Graziano, G Prota, S Truffa, D Vincenti, R Rovito, M Sgarlata, Italy A Giacometti, A Costantini, V Barocci, A Saracino, C Santoro, E Milano, L Comi, C Suardi, P Viale, L Badia, S Cretella, EM Erne, A Pieri, E Quiros Roldan, E Focà, B Menzaghi, C Abeli, L Chessa, F Pes, P Maggi, L Alessio, G Nunnari, BM Celesia, J Vecchiet, K Falasca, A Pan, S Dal Zoppo, D Segala, F Bartalesi, A Bartoloni, B Borchi, C Costa, S Lo Caputo, S Ferrara, M Bassetti, E Pontali, S Blanchi, N Bobbio, C. Del Borgo, R. Marocco, G. Mancarella, S Piconi, C Molteni, S Rusconi, G Canavesi, G Pellicanò, G Marchetti, S Antinori, A Gori, M Puoti, A Castagna, A Bandera, V Bono, MV Cossu, A Giacomelli, R Lolatto, MC Moioli, L Pezzati, S Diotallevi, C Tincati, C Mussini, M Menozzi, P Bonfanti, G Lapadula, V Sangiovanni, I Gentile, V Esposito, N Coppola, FM Fusco, G Di Filippo, V Rizzo, N Sangiovanni, S Martini, AM Cattelan, D Leoni, A Cascio, M Trizzino, D Francisci, E Schiaroli, G Parruti, F Sozio, D Messeri, SI Bonelli, C Lazzaretti, R Corsini, A Antinori, R Cauda, C Mastroianni, L Sarmati, A Latini, A Cingolani, I Mastrorosa, S Lamonica, M Capozzi, M Camici, I Mezzaroma, M Rivano Capparuccia, G Iaiani, C Stingone, L Gianserra, J Paulicelli, MM Plazzi, G d'Ettore, M Fusto, M Lichtner, I Coledan, G Madeddu, A De Vito, M Fabbiani, F Montagnani, A Franco, R Fontana Del Vecchio, D Francisci, C Di Giuli, GC Orofino, G Calleri, G Di Perri, S Bonora, G Accardo, C Tascini, A Londero, G Battagin, S Nicolè, G Starnini, S Dell'Isola

**Affiliations:** ^1^ Department of Epidemiology Istituto Nazionale per le Malattie Infettive “L. Spallanzani” IRCCS Rome Italy; ^2^ Fondazione ICONA Milan Italy; ^3^ National PhD Programme in One Health approaches to infectious diseases and life science research, Department of Public Health, Experimental and Forensic Medicine University of Pavia Pavia Italy; ^4^ Infectious and Tropical Diseases Unit IRCCS San Raffaele Milan Italy; ^5^ Department of Medicine Fondazione IRCCS Ca' Granda Ospedale Maggiore Policlinico Milan Italy; ^6^ ASST Santi Paolo e Carlo, Department of Health Science University of Milan Milan Italy; ^7^ Department of Medical and Surgery Sciences Fondazione Policlinico Universitario Agostino Gemelli IRCCS Rome Italy; ^8^ Department of Medicine University of Palermo Palermo Italy; ^9^ Centro di Riferimento Oncologico (CRO) IRCCS Aviano Italy

**Keywords:** AIDS, cancers, HIV, incidence, risk

## Abstract

The cancer risk of people with HIV (PWH) enrolled in the Italian COhort of Naives to Antiretrovirals (ICONA) with HIV diagnosis occurred between 01/1997 and 12/2023 has been compared to that of the corresponding general population of Italy. Incident cancers were grouped according to their association with viral infections. Standardized incidence ratios (SIRs) were estimated as the ratio between the observed number of cancers among PWH and the expected ones among the general population. Competing risks cumulative incidence curves and Gray's test were used to compare incidence between groups (gender and CD4+ T‐cells values at diagnosis) with death as a competing risk. Overall, 17,298 PWH (79.1% males) contributing to 133,851 person‐years of follow‐up were included; 763 (4.4%) developed > = 1 incident cancers, the most frequent cancers being KS (*N* = 204), NHL (*N* = 127) and lung‐trachea‐bronchus (*N* = 66). PWH were at 1.6‐times higher risk for all cancers compared to the general population (SIR = 1.6, 95% CI: 1.5–1.8), with significantly increased SIR for almost all virus‐related cancers, including KS (SIR = 145), NHL (SIR = 5.8), ICC (SIR = 6.0), anal cancer (SIR = 21.0) and Hodgkin lymphoma (SIR = 10.0). Apart from lung cancer risk (SIR = 1.3), none of the non‐virus‐related cancers turned out to be more frequent among PWH. PWH with CD4 + <200 cells/mm^3^ at cART initiation showed the highest cancer incidence, 5% after 5 years, versus 3% for CD4+ 200–349 and 2% for CD4+ >350 (*p* < .001). These findings underscore the need to continue prevention efforts in PWH, including behavioral risk reduction, early cART initiation, screening, and vaccination.

## Graphical abstract

What's new?

Immune suppression induced by HIV infection significantly increases the risk of certain cancers among people with HIV (PWH). In the present study, the authors investigated cancer incidence among HIV‐infected individuals in Italy using data from the Italian Cohort Naïve Antiretrovirals. Comparison with the corresponding general population in Italy over the past 25 years reveals excess cancer incidence among PWH, despite the availability of combination antiretroviral therapy to control HIV. The most frequently occurring cancers in PWH included Kaposi sarcoma, non‐Hodgkin lymphoma, and cancers of the lung–trachea–bronchus. The findings underscore the need for tailored cancer prevention strategies for PWH in Italy.

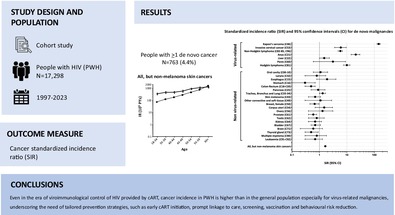

**Abbreviations**
ADM: AIDS‐defining malignancies
AIDS: acquired immune deficiency virus
cART: combined antiretroviral therapy
HCC: hepatocellular carcinoma
HL: Hodgkin lymphoma
ICC: invasive cervical cancer
ICONA: Italian Cohort of naïves to antiretrovirals
KM: Kaplan Meier
KS: Kaposi's sarcoma
NADM: non AIDS‐defining malignancies
NHL: non‐Hodgkin lymphoma
PWH: people living with HIV
SIR: standardized incidence ratio

## 1 INTRODUCTION

Human Immunodeficiency virus (HIV) infection is associated with an increased risk of several cancer types that often manifest with an aggressive clinical course and poor prognosis.^1^ In addition to three malignancies that are included in the AIDS definition, that is, Kaposi sarcoma (KS), non‐Hodgkin lymphomas (NHL), and invasive cervical cancer (ICC),^2^ several other cancer types occur at a higher frequency among people with HIV (PWH).^3^ These cancers are mostly related to chronic viral infections, such as Epstein Barr Virus‐related cancer (Hodgkin lymphoma—HL, NHL), HPV‐related cancers (anal cancer, vulvar, vaginal, penile cancer), HBV‐ and HCV‐related cancer (liver cancer), or to exposure to well‐established individual risk factors (e.g., tobacco use and lung cancer or consumption of alcoholic beverages and liver cancer).^4^

It is well known that HIV‐induced immune suppression plays a key role in increasing the cancer risk of PWH, particularly for virus‐related cancers, and risk has been shown to increase with low CD4 cell count, high HIV viral load, and a prior AIDS diagnosis.^5^ Following the introduction of combined antiretroviral therapy (cART), the natural course of HIV infection has dramatically changed, with a strong decline in the frequency of AIDS‐defining conditions including AIDS‐defining malignancies (ADM), and considerable improvements in the life expectancy of PWH.^6^ Conversely, also in the ART era non‐AIDS defining malignancies (NADMs) represent the most frequent condition^7^ and the second most common cause of death.^6^ The risk of NADM in PWH is multifactorial and particularly impacted by highly prevalent oncogenic viral co‐infections and smoking.^8^ Another important driver is identified in HIV‐induced immune activation and persistent inflammation.^9^ Chronic immune activation can persist even among virally suppressed PWH, leading to an exhaustion of the immune resources, mimicking the process of aging‐associated immune senescence, and installing an immune dysfunction.^10^

In a recent review, it was proposed that the terms ADMs along with the opposing one NADMs should be considered obsolete, suggesting to consider each cancer type arising in PWH individually, with a particular focus on tumors associated with virus (e.g., anal cancer and Hodgkin lymphoma).^11^

Currently, several studies, up to date, compared Europe and North America^12^, ^13^ the cancer incidence among PWH and the general population, but no recent investigations were carried out in Italy.

This study aimed to assess the cancer risk of PWH enrolled in the multicentric Italian cohort study ICONA and to compare it with the age and sex corresponding to the Italian general population.

## 2 METHODS

### 2.1 Study population

The Italian Cohort Naïve Antiretrovirals (ICONA) is an ongoing national HIV cohort set up in 1997 in Italy, enrolling antiretroviral (ART) naïve persons with HIV (PWH) from 57 infectious diseases centers whose Institutional Review Boards approved the ICONA Foundation Study protocol.^14^, ^15^ PWH naïve from ART are enrolled after providing written informed consent to study participation and processing of personal data, according to the Helsinki Declaration.

After enrolment, at least 2 study visits are being conducted every year to collect sociodemographic, clinical, and laboratory data using medical records through a standardized protocol (www.icona.org): smoke, drug use, body weight, height, blood pressure, AIDS diagnosis, details on antiretroviral therapy used and reasons for changes, non‐AIDS comorbidities, comedication, hospitalization, and instrumental diagnostic evaluation are collected at every visit. All available HIV‐related and laboratory results are also collected at each visit: virology, immunology, chemistry, hematology, and serologies on viral co‐infections. HIV genotype resistance tests, HCV and HBV genotypes, and viral load are collected when available. Furthermore, additional liver diagnostic information (fibroscan, liver pathologies, HBV vaccination, and treatments) is collected. Dates of loss to follow‐up and of death are collected together with the cause of death using the Coding of Death format.

All PWH enrolled in the ICONA cohort with HIV diagnoses that occurred between January 1997 and December 2023 were included in this analysis. Data collected included demographic and socio‐behavioral indicators at the time of enrolment; plasma HIV‐RNA and CD4 cell count at enrolment and at each clinical visit (on an average, every 4–6 months), and clinical events both HIV‐ or non‐HIV‐related. The study period spanned from 30 days after the first HIV diagnosis to the date of the occurrence of cancer, the last clinical visit, or death. For this reason, individuals with follow‐up shorter than 30 days from HIV diagnosis were excluded from this analysis.

For the purpose of this analysis, all cancer diagnoses were histologically confirmed and coded according to the International Classification for Disease and Related Health Problems 10th revision (ICD‐10). A cancer diagnosis received before HIV diagnosis or those that occurred within the first 30 days after HIV diagnosis were considered as prevalent cancer cases, and they were excluded from the present analysis of the incidence of de novo malignancies after HIV diagnosis. In situ carcinomas and other pre‐neoplastic lesions (ICD‐O‐2 behavior codes 0–2) were also excluded as well as recurring and metastatic cancers and non‐melanoma skin cancers.

Incident (de novo) cancers were further grouped in virus‐related malignancies (i.e.,: KS, NHL, ICC, Hodgkin lymphoma—HL, anal, liver, vulvar, penile cancer and Merkel cell carcinoma), non‐virus related malignancies, and those not otherwise specified cancers. Multiple primary tumors were separately analyzed in site‐specific sites. Supplementary Table S1 shows the ICD‐10 codes used to define each cancer site or specific group.

### 2.2 Statistical analysis

For each patient, person‐years (PYs) at risk of cancer were computed from 30 days after the first HIV‐positive test to the date of cancer diagnosis, date of death, or date of last follow‐up, whichever occurred first.

After a cancer diagnosis, patients no longer contribute to the determination of person‐time at risk of that specific cancer type. However, we did not censor these individuals at their first cancer diagnosis, because they could still be at risk of developing other types of cancer, continuing to add follow‐up time to the determination of PYs at risk for further specific tumor.

To determine absolute risk estimates of de novo malignancies, cumulative incidence functions were estimated using a competing risk approach with nonparametric indicators, with death as a competing event.

We computed age‐specific cancer incidence rates for major cancer types and plotted them against the age‐specific incidence of the specific cancer type among the corresponding general Italian population.

Standardized incidence ratios (SIRs) were estimated as the ratio between the observed number of cancer cases among cohort members and the expected one among the corresponding general population of Italy. The expected number of cases was computed by multiplying the PYs at risk among cohort members with sex, age (5‐year groups), residence area, and calendar period‐specific incidence rates from all Italian cancer registries as published by the International Agency for Cancer (IARC) in Cancer Incidence in Five Continents, vol. VIII (1993–1997), vol. IX (1998–2002), vol. X (2003–2007), vol. XI (2008–2012) and vol. XII (from 2013 thereafter). Corresponding 95% confidence intervals (CIs) for SIRs were calculated assuming a Poisson distribution.

A further analysis was conducted to evaluate the cumulative incidence of cancers after cART initiation, considering death as a competing risk, using Gray's test to compare CD4+ T‐cell categories at cART. In this analysis, similarly to what was performed in the general incidence analysis, incident cancers were considered all malignancies diagnosed at least 30 days or beyond cART initiation.

A two‐tailed p‐value<0.05 was considered statistically significant. Statistical analyses were performed using Stata release 17 (StataCorp LLC, 2021; College Station, TX), R version 4.4 (R Foundation for Statistical Computing, Vienna, Austria), or SPSS version 29 (IBM Corp. Released 2023. Armonk, NY: IBM Corp).

## 3 RESULTS

Overall, 17,298 PWH (79.1% males) were included, contributing to a total of 133,851 PYs of follow‐up (median follow‐up 6.6 years; Interquartile range, IQR: 2.7–11.1) (Table 1). The median age at HIV diagnosis was 37 years in males and 35 years in females, and most of the cohort members acquired HIV infection through sexual intercourse: among males, 56.3% reported sexual relations with other men (MSM) and 29.5% sexual relations with females; 85.2% of females turned out to have acquired HIV infection through heterosexual intercourse. At the time of inclusion in the study group, 47.6% of cohort members had as a first CD4+ cell count less than 350 CD4+ cells/mm^3^ (46.5% in males, and 51.6% in females), while 9.9% were diagnosed with AIDS (Table 1). 543 (3.1%).

**TABLE 1:** General characteristics of 17,298 people living with HIV enrolled in the ICONA Cohort Study: Italy, 1997–2023.

	Total	Male	Female
	*N* (%)	*N* (%)	*N* (%)
All	17,298 (100.0)	13,680 (79.1)	3618 (20.9)
Age at HIV‐diagnoses (years)			
18–24	1762 (10.2)	1303 (9.5)	459 (12.7)
25–34	5909 (34.2)	4558 (33.3)	1351 (37.3)
35–44	5101 (29.5)	4150 (30.3)	951 (26.3)
45–54	2900 (16.8)	2373 (17.4)	527 (14.6)
55+	1626 (9.4)	1296 (9.5)	330 (9.1)
Calendar period of HIV‐diagnoses			
1997–2008	4513 (26.1)	3271 (23.9)	1242 (34.3)
2008–2012	3766 (21.8)	3035 (22.2)	731 (20.2)
2013–2017	5061 (29.3)	4111 (30.1)	950 (26.3)
2018–2023	3958 (22.9)	3263 (23.9)	695 (19.2)
Mode of HIV acquisition			
Men who have sex with men	7695 (44.5)	7695 (56.3)	0 (−)
Heterosexual intercourse	7112 (41.1)	4031 (29.5)	3081 (85.2)
Use of intravenous drugs	1338 (7.7)	1056 (7.7)	282 (7.8)
Other/unknown	1153 (6.7)	898 (6.6)	255 (7.1)
First CD4+ count/mm^3^			
<200	4974 (28.8)	3829 (28.0)	1145 (31.7)
200–349	3246 (18.8)	2526 (18.5)	720 (19.9)
350–499	3489 (20.2)	2840 (20.8)	649 (17.9)
500+	5518 (31.9)	4435 (32.4)	1083 (29.9)
*Unknown*	*71* (*0.4*)	*50* (*0.4*)	*21* (0.*6*)
AIDS diagnosis at enrolment:			
No	15,578 (90.1)	12,362 (90.4)	3216 (88.9)
Yes	1720 (9.9)	1318 (9.6)	402 (11.1)
Patients with prevalent cancers at enrolment	543 (3.1)	441 (3.2)	102 (2.8)
Person‐years of follow‐up			
Total	133,851	10,366	30,185
Median (inter quartile range)	6.6 (2.7–11.1)	6.5 (2.7–10.9)	6.9 (2.8–11.9)
Patients with one or more cancer^a^ diagnoses at follow‐up	763 (4.4)	624 (4.6)	139 (3.8)
Total cancer^a^ diagnoses	789	641	148

^a^ *De novo* malignancies diagnosed at least 30 days after HIV diagnosis, excluding non‐melanoma skin cancers.

During the follow‐up, a total of 789 incident cancer diagnoses were documented in 763 PWH (4.4%) (Table 1). As shown in Table 1 and in Supplementary Table S1, KS (204 cases, 192 in males) was the most frequent cancer, followed by NHL (127 cases, 109 of which in males). Among other virus‐related cancers, 57 cases of Hodgkin lymphoma were reported, 32 anal, 25 liver cancer, and 18 ICC. Conversely, among the remaining non‐virus‐related malignancies, the most frequent were trachea, bronchus, and lung cancers (66 cases, 52 in males), followed by prostatic cancer (36 cases), female breast cancer (29 cases) and bladder cancers (28 diagnoses) (see Table 2 and Supplementary Table 1).

**TABLE 2:** Standardized incidence ratios (SIR) and 95% confidence intervals (CI) for incident cancers according to gender.

	ICD‐10 codes^a^	No. of cancer cases		SIR (95% CI)	No. of cancer cases	SIR (95% CI)	
Type/site		Observed	Expected		Male/Female	Male	Female
Virus‐related							
Anus	C21	32	1.5	**21.0 (14.4–29.7)**	31/1	**27.6 (18.7–39.2)**	NC
Liver	C22	25	16.4	*1.5 (1.0–2.3)*	21/4	1.4 (0.9–2.1)	**3.8 (1.0–9.7)**
Kaposi's sarcoma	C46	204	1.4	**144.8 (125.6–166.1)**	192/12	**141.6 (122.2–163.1)**	**230.0 (118.9–401.8)**
Invasive cervical cancer	C53	18	3.0	**6.0 (3.6–9.5)**	−/18	‐	**6.0 (3.6–9.5)**
Penis	C60	3	1.0	3.1 (0.6–9.0)	3/−	3.1 (0.6–9.0)	‐
Hodgkin lymphoma	C81	57	5.7	**10.0 (7.6–13.0)**	48/9	**10.4 (7.7–13.8)**	**8.3 (3.8–15.8)**
Non‐Hodgkin Lymphomas (All types)	C82‐85, C96	127	21.9	**5.8 (4.8–6.9)**	109/18	**5.9 (4.9–7.1)**	**5.1 (3.0–8.1)**
Non virus‐related							
Oral cavity	C00‐10	11	9.8	1.1 (0.6–2.0)	10/1	1.1 (0.5–2.1)	NC
Esophagus	C15	7	3.6	1.9 (0.8–4.0)	5/2	1.5 (0.5–3.4)	NC
Stomach	C16	5	16.6	0.3 (0.1–0.7)	5/0	**0.3 (0.1–0.8)**	‐
Colon‐rectum	C18‐20	24	51.3	**0.5 (0.3–0.7)**	21/3	**0.5 (0.3–0.8)**	*0.4 (0.1–1.1)*
Pancreas	C25	12	12.9	0.9 (0.5–1.6)	8/4	0.7 (0.3–1.5)	2.0 (0.5–5.1)
Larynx	C32	6	7.9	0.8 (0.3–1.7)	5/1	0.7 (0.2–1.5)	NC
Trachea, bronchus and lung	C33‐34	66	50.3	**1.3 (1.0–1.7)**	52/14	1.2 (0.9–1.5)	2.6 (1.4–4.3)
Skin melanoma	C43	19	27.7	0.7 (0.4–1.1)	14/5	0.6 (0.4–1.1)	0.8 (0.3–1.9)
Other connective and soft tissue	C49	4	3.7	1.1 (0.3–2.8)	4/0	1.3 (0.4–3.4)	‐
Breast, female	C50	29	43.3	**0.7 (0.5–1.0)**	−/29	‐	**0.7 (0.5–1.0)**
Corpus uteri	C54	8	5.2	1.5 (0.7–3.0)	−/8	‐	1.5 (0.7–3.0)
Ovary	C56	5	3.8	1.3 (0.4–3.1)	−/5	‐	1.3 (0.4–3.1)
Prostate	C61	36	61.5	**0.6 (0.4–0. 8)**	36/−	**0.6 (0.4–0.8)**	‐
Testis	C62	9	11.5	0.8 (0.4–1.5)	9/−	0.8 (0.4–1.5)	‐
Kidney	C64	15	20.6	0.7 (0.4–1.2)	14/1	0.8 (0.4–1.3)	NC
Bladder	C67	28	38.1	0.7 (0.5–1.1)	27/1	0.7 (0.5–1.1)	NC
Brain	C71	6	9.9	0.6 (0.2–1.3)	4/2	0.5 (0.1–1.2)	NC
Thyroid gland	C73	11	21.3	**0.5 (0.3–0.9)**	6/5	0.5 (0.2–1.1)	0.5 (0.2–1.2)
Multiple myeloma	C90	5	6.4	0.8 (0.3–1.8)	3/2	0.6 (0.1–1.6)	NC
Leukemia	C91‐92	8	12.1	0.7 (0.3–1.3)	7/1	0.7 (0.3–1.4)	NC
All, but non‐melanoma skin cancers^b^, ^c^		789	479.4	**1.6 (1.5–1.8)**	641/148	**1.7 (1.6–1.9)**	**1.4 (1.2–1.6)**

*Note*: SIR that are statistically significant are highlighted in bold (*p*‐value <.05) or italic (*p*‐value <.10). NC: not calculated if less than 3 cases. ICONA Cohort Study: Italy, 1997–2023.

^a^ ICD‐10: International Classification of Diseases, tenth revision.

^b^ Sites/types with <3 observed cases and those not otherwise specified cancers are not shown in the table (see Supplementary Table S1 for details).

^c^ Non‐melanoma skin cancers excluded.

**FIGURE 1:**
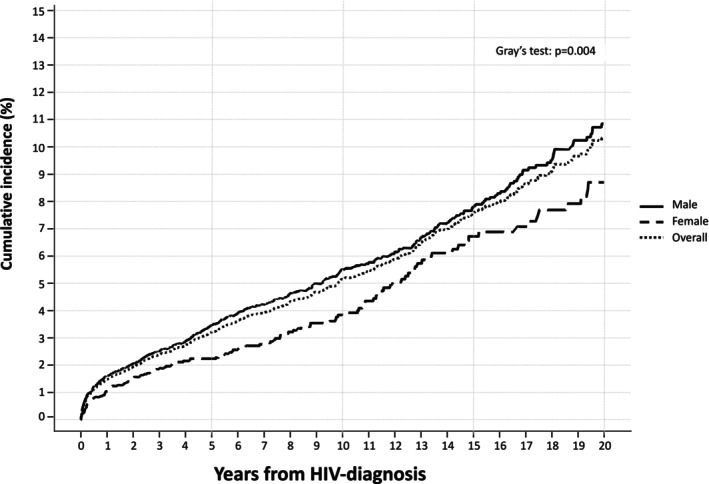
Cumulative incidence of de novo malignancies (not including skin non‐melanoma cancers), according to diagnostic group and time since first HIV‐positive test: ICONA Cohort Study: Italy, 1997–2023.

Figure 1 illustrates the cumulative incidence of cancer according to the time elapsed from the first HIV‐positive test in total and according to gender, showing that overall, roughly 10% of the PWH included in the analysis were diagnosed with cancer up to 20 years of observation. Males present a cumulative incidence of cancer higher than females, in particular in the long run (2.6% vs. 1.9% in the first 3 years from HIV diagnosis, 10.8% vs. 8.8% at 20 years). Supplementary Figure S1 shows the timing of type of cancer diagnoses according to period, showing that Kaposi's sarcoma incident cases hugely decreased over time, with 62 cases diagnosed in the first period (1997–2008; which represented 39% of de novo malignancies diagnosed in the period) to 48 cases in the last period (2018–2023; 19% proportion). It is to be noted conversely that non‐virus‐related malignancies concurred to 31% of de novo cancers in the first period, increasing to 54% in the last one (Supplementary Table S1).

**FIGURE 2:**
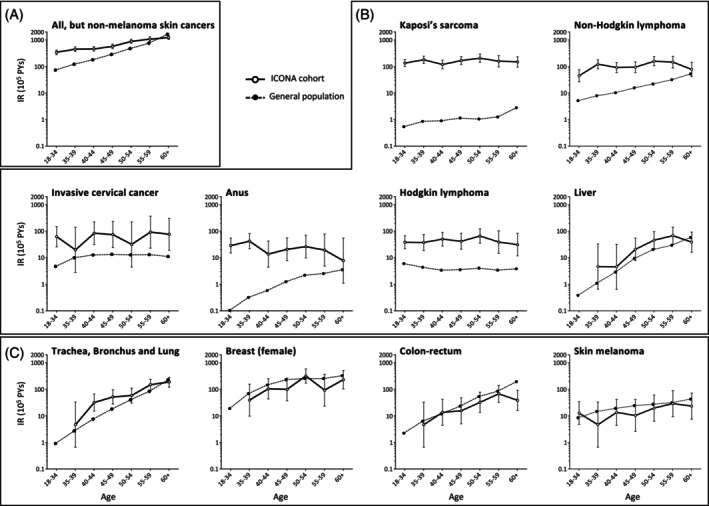
Comparison of age specific cancer incidence rates by cancer types in the ICONA cohort versus the general population. Panel (A): All, but non‐melanoma skin cancers; panel (B): Virus‐related malignancies; panel (C): Non‐virus related malignancies.

Figure 2 compares age‐specific incidence rates observed in PWH with those expected in the corresponding general population. In general (panel A) PWH showed significantly higher incidence for all age categories at least up to 54 years of age, while for those 55 or older, PWH, the risk was comparable. When considering virus‐related cancers, incidence rates were higher in PWH than in the general population for Kaposi's sarcoma, invasive cervical cancer, and Hodgkin lymphoma in all age groups, while for NHL and anal cancers, the risk generally higher showed a tendency to equivalence only in the older age group (60 or more years of age) (panel B). In contrast, no difference in terms of incidence over age was observed for more frequent cancers in the non‐virus‐related malignancies group (panel C) such as lung, breast female, colon‐rectum, or skin melanoma (see Figure 2).

**FIGURE 3:**
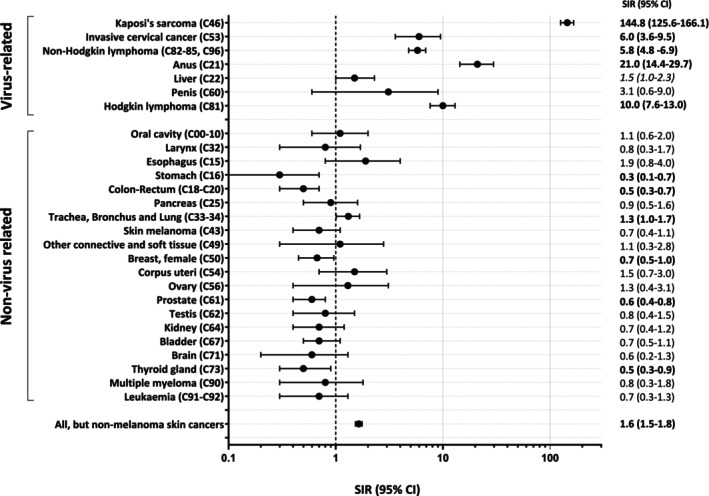
Standardized incidence ratios (SIR) and 95% confidence intervals (CI) for incident cancers. ICONA.

As compared to the corresponding general population, PWH in our study group turned out to be at 1.6 times higher risk for all cancers (SIR = 1.6, 95% CI: 1.5–1.8) (Table 2). Increased SIRs were noted for virus‐related cancers and in particular for Kaposi's sarcoma (SIR = 145, 95% CI: 126–166; higher in females, SIR = 230), NHL (SIR = 5.8, 95% CI: 4.8–6.9), Hodgkin lymphoma (SIR = 10.0, 95% CI: 7.6–13.0), anal cancer (SIR = 21.0, 95% CI: 14.4–29.7) and ICC (SIR = 6.0, 95% CI: 3.6–9.5). In non‐virus‐related cancers, statistically increased risks were noted only for trachea, bronchus, and lung cancer (SIR = 1.3, 95% CI: 1.0–1.7), with a risk 2.6‐fold significantly increased in females (95% CI: 1.4–4.3). Conversely, significantly decreased risks were observed for stomach cancer (SIR = 0.3), colon‐rectum (SIR = 0.5), breast (female, SIR = 0.7), prostate (SIR = 0.6), and thyroid cancer (SIR = 0.5). An overall picture showing SIR estimation according to type of cancer is depicted in Figure 3.

**FIGURE 4:**
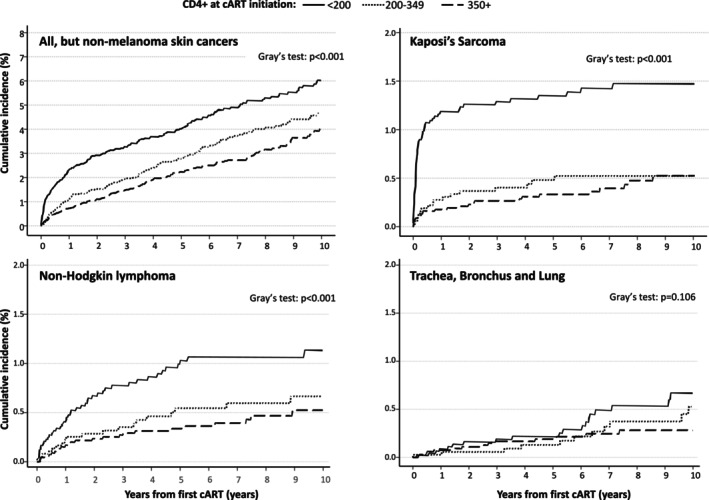
Cumulative incidence of malignancies since initiation of combination antiretroviral therapy (cART)according to CD4+ count. ICONA Cohort Study: Italy, 1997–2023.

PWH with CD4+ cell count < 200/mm^3^ at cART initiation showed the highest cumulative cancer incidence, about 4% after 5 years from first cART, as compared to 3% of those with CD4 cell counts between 200 and 349/mm^3^ at cART initiation and 2% of those with CD4+ cell counts >350/mm^3^ (Gray's test *p* < .001). A similar picture emerged in PWH who developed KS and NHL (Figure 4). No difference in cumulative incidence of trachea, bronchus, and lung cancer according to CD4+ count at cART initiation (Gray's test *p* = .106).

## 4 DISCUSSION

The results of this cohort study show that over the past 25 years, despite the survival successes that ART has enabled, one out of 10 PWH enrolled in the ICONA cohort, accounting for more than 20,000 PWH, was diagnosed with cancer. Overall, the most frequent cancer observed was KS and NHL among the virus‐related cancers, and trachea/bronchus/lung among non‐virus‐related ones. Nevertheless, if the different periods were considered, an increased proportion of non‐virus‐related cancers and a strong reduction of virus‐related ones were observed over the years. An excess incidence of cancer in PWH compared to what is observed in the general population has been observed for most of the cancers included in the analysis (cumulative SIR 1.6), remarkably for all virus‐related cancers.

Regarding virus‐related cancers, rates of KS, NHL, and cervical cancer have declined sharply in developed countries during the highly active antiretroviral therapy era,^2^ and a similar decline has been observed even in the present analysis; nevertheless, their incidence ratios remain elevated compared with the general population, as previously reported.^2^ The relative increased risk of virus‐related cancers compared to nonvirus‐related malignancies observed in the first 5 years of observation could be interpreted as an increased risk still associated with the presence of immunological dysregulation in the first years of cART; a switch in incidence toward virus‐related non‐AIDS malignancies could be due to competitive oncogenic risk in the long run on which the effect of ART is not well defined.^16^, ^17^

In a recent meta‐analysis of studies published between 1992 and 2022, nearly all types of the 20 virus‐related non‐AIDS defining cancers occurred at increased rates among PWH in comparison with the general population, as analyzed through standard incidence rates (SIR). The highest SIRs were observed in HPV‐related cancers of the anogenital sites, including cancers of the anus, anal canal, and penis. Cancers with markedly increased rates also included Hodgkin lymphoma, neck cancer, and liver cancer, which are related to Epstein–Barr virus (EBV), human papillomavirus (HPV), and hepatitis B/C virus (HBV/HCV) respectively.^18^ In our study, a highest incidence ratio for anal (SIR 21.0, 27.6 in male population), Hodgkin's disease (SIR 10.0), ICC (SIR = 6.0) and NHL (SIR = 5.8) was observed. Data on anal cancer suggest that screening in the HIV‐infected population, despite being indicated in all guidelines,^19^, ^20^ should be reinforced. It has not yet penetrated so far into clinical practice to date, and the high incidence reported in our study may certainly be influenced by the large period of our cohort and the total absence of guidelines in the earlier years. Data from NA‐ACCORD documented a decline in the risk of anal cancer of 2.2% per year in the North American PWH from 2001 to 2016, varying by geographic area, closely correlated with the prevalence of screening in clinical practice.^21^

The excess of Hodgkin's disease in PWH over that observed in the general population found in our study is consistent with that reported in a recent meta‐analysis. The 10‐fold increased risk suggests, as in the case of anal cancer, that the correlation with viral oncogenesis, EBV‐related in this case, keeps the incidence of this neoplasm high in PWH despite the viroimmunological control achievable with modern cART.^22^

Looking at incidence rates according to age, an overall significantly higher incidence of cancers compared to the general population has been observed in this study for all age categories at least up to 54 years of age, while for those 55 or older the risk was comparable. Nevertheless, considering virus‐related cancers, incidence rates were higher in PWH than in the general population for KS, invasive cervical cancer, and Hodgkin lymphoma in all age groups, while for NHL and anal cancers, the risk showed a tendency to equivalence only in those more than 60 years old. On the contrary, no difference in terms of incidence over age was observed for non‐virus‐related cancers.

This result is partially in line with previous studies showing an earlier age at cancer diagnosis for some cancers, in particular for lung cancer, anal cancer, and myeloma.^23^, ^24^, ^25^ Another study, using the French Hospital Database on HIV, observed younger ages at diagnosis for lung, but not anal cancer.^26^

One possible explanation for younger ages at the diagnosis for certain cancers in PWH is that HIV may accelerate the time from the initiation of the carcinogenic process, or it could be due also to earlier or stronger exposure to key risk factors in PWH,^27^ such as smoking, HPV persistent infection among men who have sex with men (MSM), the risk group with the highest anal cancer risk,^28^ or, in turn, an increased prevalence of anal Pap testing in PWH, particularly MSM.^29^

Our analysis showed a slightly increased risk of liver cancer (SIR 1.5, *p*‐value<.10) in PWH compared to the general population, lower compared to what has been reported in other studies.^18^ It is well known that prevention of liver cancer includes treatment of the Hepatitis B virus and hepatitis C infection, as well as controlling metabolic and behavioral risk factors associated with liver cancer (diabetes, hypertension, cardiovascular diseases, potus). Italy is one of the leading countries in terms of liver cancer incidence overall; for this reason, the relative risk should be lower than observed in other cohorts.^30^ Moreover, HBV vaccination has been associated with reductions in HCC incidence, and in Italy, HBV vaccination has been widely spread among PWH as a component of routine monitoring of HIV infection, and, besides HBV vaccines, treating chronic hepatitis B with nucleoside analogs has been included in most PWH as a component of cART during the years of the cohort. For HCV, the treatment of existing HCV infections with direct‐acting antiviral (DAA) therapies, being the most effective way to prevent new cases of HCC, has been widely used in PWH in Italy, as demonstrated by ICONA cohort data.

According to sex, it is worth mentioning a high SIR for trachea/bronchus/lung emerged among females, suggesting a higher frequency of tobacco smoking among HIV‐infected young adult females than in their counterparts in the general population.

A lower incidence of breast cancer among females with HIV compared with the general population was also found. Previous studies proposed that it might be because HIV could bind to the chemokine receptor CXCR4 expressed by breast cancer cells, resulting in apoptosis.^31^, ^32^

Consistent with other studies, we did not observe overall differences by HIV status in the risk of non‐infectious related cancers,^33^ except for trachea, bronchi, and lung (SIR 1.5). This is largely^34^ linked to the higher proportion of smokers in PWH than in the general population, potentially contributing to a higher incidence of cancers induced by smoking.^35^

The immunological implications that can be derived from the results of our study are two. First, the highest incidence of cancers was observed in PWH with CD4 cell counts of less than 200/mm^3^. This finding is further confirmation of the fact that immunological deficiency is a risk factor for the development of both AIDS and non‐AIDS related events and in particular neoplasms; in a recent cohort study in Ontario, from 1997 to 2020, among 4771 PWH, low baseline CD4 (<200 cells/mm^3^), nadir below 200 cells/mm^3^, low time‐updated CD4, and time‐updated CD4:CD8 ratio were associated with an increased rate of infection‐related cancers.^36^ These results further reinforce the need for early diagnosis and linkage to care and high antiretroviral therapy uptake as a pivotal strategy combined with oncological screening and vaccine uptake to reduce the cancer incidence in PWH.

Second, HIV infection leads to chronic immune activation/inflammation that can persist in virally suppressed persons on cART, increasing the risk of malignancies both virus‐related and non‐virus‐related.^8^ The CD4:CD8 ratio represents a surrogate marker of defective T lymphocytes and enhanced immune activation in HIV infection and may characterize a subpopulation with distinct immunological abnormalities and chronic inflammation.^37^ Recently, a lower CD4:CD8 ratio (<0.5) has been shown in a large multi‐cohort study across Europe and Australia to be associated with an increased risk of several cancers.^38^

Our study has some limitations. First of all, although ICONA is a cohort strongly representative of the reality of PWH in Italy, it is not the equivalent of a national surveillance system in terms of the extent of events collection; secondly, ICONA is representative of PWH linked to care, therefore inferences on the incidence of neoplasms in PWH is limited only by considering linked to care individuals. Nevertheless, it must be considered that the number and the characteristics of new diagnoses of HIV infection in PWH enrolled in the ICONA cohort in recent years are in line with what has been reported at the Italian level by the registry data of the “Istituto Superiore di Sanità,” demonstrating that ICONA is relevantly representative of the entire population living with HIV in Italy.^15^ Third, our study investigates the role of HIV status as the total effect of it, without dissecting whether the different risk compared to the general population is led by HIV infection itself and its effects (immunodeficiency, inflammation, long‐term antiretroviral toxicities) or by the different behaviors of HIV individuals. Fourth, by using the SIRs we have accounted for sex, age, residence area, and calendar period, but other residual confounding cannot be ruled out.

In conclusion, in this cohort study covering the last 25 years of HIV infection, the incidence risk of cancers in PWH is still remarkably high compared to that in the general population. Our results further confirm the need to implement prevention strategies in people with HIV both related to the direct management of HIV infection (early initiation of antiretroviral therapy and linkage to care), periodic screening and vaccination for diseases for which a vaccine is available, and behavioral risk reduction strategies toward environmental oncogenes. Furthermore, based on the results of our and other studies, it could be advisable, for public health reasons, to consider virus‐related NADMs, namely anus and penile HPV‐related cancer and Hodgkin lymphoma, as AIDS‐defining illnesses, similar to cervical cancers in females.

## AUTHOR CONTRIBUTIONS

**Pierluca Piselli:** Conceptualization; data curation; formal analysis; methodology; software; writing – original draft. **Alessandro Tavelli:** Data curation; formal analysis; project administration; software. **Claudia Cimaglia:** Data curation; formal analysis; software; writing – original draft. **Camilla Muccini:** Investigation. **Alessandra Bandera:** Conceptualization; investigation. **Giulia C. Marchetti:** Investigation; supervision; writing – review and editing. **Carlo Torti:** Supervision; writing – review and editing. **Valentina Mazzotta:** Investigation. **Luca Pipitò:** Investigation. **Alessandro Caioli:** Formal analysis. **Enrico Girardi:** Methodology; supervision. **Andrea Antinori:** Funding acquisition; supervision; writing – review and editing. **Diego Serraino:** Formal analysis; methodology; supervision; writing – original draft. **Antonella d'Arminio Monforte:** Funding acquisition; project administration; supervision; writing – review and editing. **Antonella Cingolani:** Conceptualization; methodology; supervision; writing – original draft.

## FUNDING INFORMATION

The present study did not receive any funding. The ICONA Foundation is supported by unrestricted grants from ViiV Healthcare, Gilead Sciences, MSD. The funders of the ICONA Foundation had no role in the study design, data collection, analysis, decision to publish, or preparation of this study.

## CONFLICT OF INTEREST STATEMENT

Enrico Girardi received a research grant from Gilead Sciences and speaker fees from ViiV healthcare and Gilead Sciences. Andrea Antinori served as paid consultant for AstraZeneca, Gilead, MSD, Janssen‐Cilag, GSK, Moderna, Pfizer, Bavarian Nordic, ViiV Healthcare and received institutional grants from Gilead, AstraZeneca, ViiV Healthcare. Alessandra Bandera reported a potential conflict of interest with Gilead (grant for data publication), AstraZeneca, Biomerieux, Qiagen, Janssen‐Cilag, Nordic Pharma, Pfizer, ViiV, Sobi, Angelini Pharma. The other authors declare no conflict of interest related to the present manuscript.

## ETHICS STATEMENT

The ICONA Foundation study was first approved by the local ethics committees of participating clinical sites. The last amendment has been centrally approved by the Comitato Etico Territoriale Lazio Area 4 (n 83‐2024 of 01 July 2024). All patients signed a consent form for study participation and processing of data by the ethical standards of the committee on human experimentation and the Helsinki Declaration (last amended in October 2013).

## Supporting information


**Data S1:** Supplementary Information

## Data Availability

The data that support the findings of this study are available from the corresponding author upon reasonable request.

## APPENDIX A ICONA FOUNDATION STUDY GROUP

BOARD OF DIRECTORS: A d'Arminio Monforte (President), A Antinori (Vice‐President), S Antinori, A Castagna, R Cauda, G Di Perri, E Girardi, R Iardino, A Lazzarin, GC Marchetti, C Mussini, E Quiros‐Roldan, L Sarmati, B Suligoi, F von Schloesser, P Viale.

SCIENTIFIC SECRETARY: A d'Arminio Monforte, A Antinori, A Castagna, F Ceccherini‐Silberstein, A Cingolani, A Cozzi‐Lepri, A Di Biagio, E Girardi, A Gori, S Lo Caputo, G Marchetti, F Maggiolo, C Mussini, M Puoti, CF Perno, C Torti.

STEERING COMMITTEE: A Antinori, A Bandera, S Bonora, A Calcagno, D Canetti, A Castagna, F Ceccherini‐Silberstein, A Cervo, A Cingolani, P Cinque, A Cozzi‐Lepri, A d'Arminio Monforte, A Di Biagio, R Gagliardini, A Giacomelli, E Girardi, N Gianotti, A Gori, G Guaraldi, S Lanini, G Lapadula, M Lichtner, A Lai, S Lo Caputo, G Madeddu, F Maggiolo, V Malagnino, G Marchetti, A Mondi, V Mazzotta, C Mussini, S Nozza, CF Perno, S Piconi, C Pinnetti, M Puoti, E Quiros Roldan, R Rossotti, S Rusconi, MM Santoro, A Saracino, L Sarmati, V Spagnuolo, N Squillace, V Svicher, L Taramasso, C Torti, A Vergori.

STATISTICAL AND MONITORING TEAM: A Cozzi‐Lepri, S De Benedittis, I Fanti, M Giotta, C Marelli, A Rodano’, A Tavelli.

COMMUNITY ADVISORY BOARD: M Cernuschi, L Cosmaro, A Perziano, V Calvino, D Russo, M Farinella, N Policek, VL Del Negro.

BIOLOGICAL BANK INMI AND SAN PAOLO: M Augello, S Carrara, S Graziano, G Prota, S Truffa, D Vincenti, R Rovito, M Sgarlata.

PARTICIPATING PHYSICIANS AND CENTERS: Italy A Giacometti, A Costantini, V Barocci (Ancona); A Saracino, C Santoro, E Milano (Bari); L Comi, C Suardi (Bergamo); P Viale, L Badia, S Cretella (Bologna); EM Erne, A Pieri (Bolzano); E Quiros Roldan, E Focà (Brescia); B Menzaghi, C Abeli (Busto Arsizio); L Chessa, F Pes (Cagliari); P Maggi, L Alessio (Caserta); G Nunnari, BM Celesia (Catania); J Vecchiet, K Falasca (Chieti); A Pan, S Dal Zoppo (Cremona); D Segala (Ferrara); F Bartalesi, A Bartoloni, B Borchi, C Costa (Firenze); S Lo Caputo, S Ferrara (Foggia); M Bassetti, E Pontali, S Blanchi, N Bobbio (Genova); C. Del Borgo, R. Marocco, G. Mancarella (Latina); S Piconi, C Molteni (Lecco); S Rusconi, G Canavesi (Legnano); G Pellicanò (Messina); G Marchetti, S Antinori, A Gori, M Puoti, A Castagna, A Bandera, V Bono, MV Cossu, A Giacomelli, R Lolatto, MC Moioli, L Pezzati, S Diotallevi, C Tincati (Milano); C Mussini, M Menozzi (Modena); P Bonfanti, G Lapadula (Monza); V Sangiovanni, I Gentile, V Esposito, N Coppola, FM Fusco, G Di Filippo, V Rizzo, N Sangiovanni, S Martini (Napoli); AM Cattelan, D Leoni (Padova); A Cascio, M Trizzino (Palermo); D Francisci, E Schiaroli (Perugia); G Parruti, F Sozio (Pescara); D Messeri, SI Bonelli (Pistoia); C Lazzaretti, R Corsini (Reggio Emilia); A Antinori, R Cauda, C Mastroianni, L Sarmati, A Latini, A Cingolani, I Mastrorosa, S Lamonica, M Capozzi, M Camici, I Mezzaroma, M Rivano Capparuccia, G Iaiani, C Stingone, L Gianserra, J Paulicelli, MM Plazzi, G d'Ettore, M Fusto, M Lichtner (Roma); I Coledan (Rovigo); G Madeddu, A De Vito (Sassari); M Fabbiani, F Montagnani (Siena); A Franco, R Fontana Del Vecchio (Siracusa); D Francisci, C Di Giuli (Terni); GC Orofino, G Calleri, G Di Perri, S Bonora, G Accardo (Torino); C Tascini, A Londero (Udine); G Battagin, S Nicolè (Vicenza); G Starnini, S Dell'Isola (Viterbo).
