# Supplementary material for: Cancer incidence in people with HIV in Italy: Comparison of the ICONA COHORT with general population data
Source: Int J Cancer. 2025 May 29;157(6):1142–53. doi: 10.1002/ijc.35493 (PMC12280848; doi:10.1002/ijc.35493)

CANCER INCIDENCE IN PEOPLE WITH HIV IN ITALY: COMPARISON OF THE ICONA COHORT WITH GENERAL POPULATION DATA

Pierluca Piselli, Alessandro Tavelli, Claudia Cimaglia, Camilla Muccini, Alessandra Bandera, Giulia C Marchetti, Carlo Torti, Valentina Mazzotta, Luca Pipitò, Alessandro Caioli, Enrico Girardi, Andrea Antinori, Diego Serraino, Antonella d'Arminio Monforte, Antonella Cingolani for Icona Foundation Study Group

Table of contents:
Supplementary Table S1
Supplementary Figure S1

Supplementary Table S1. Cancer sites and group definitions considered, according to the International Classification of Diseases and Related Health Problems, 10th revision (ICD-10)

Site/group name	ICD-10 code	N	M/F
**All malignancies, but non-melanoma skin-cancers**	**C00-C97 (excl. C44)**	**789**	**641/148**
**Virus-related**	**C21, C22, C4A, C46, C51, C53, C60, C81, C82-85, C96**	**469**	**406/64**
Anus	C21	32	31/1
Liver	C22	25	21/4
Merkel Cell Carcinoma (MCC)	C4A	1	1/0
Kaposi's sarcoma (KS)	C46	204	192/12
Vulva	C51	2	-/2
Invasive cervical cancer (ICC)	C53	18	-/18
Penis	C60	3	3/-
Hodgkin's lymphoma (HL)	C81	57	48/9
Non-Hodgkin lymphoma (All types. NHL)	C82-85, C96	127	109/18
**Non Virus-Related**	**C00-C97 (excl. Virus-related, C44 and C76-C80)**	**315**	**231/84**
Oral cavity	C00-10	11	10/1
Lip	C00	1	1/0
Tongue	C01	1	1/0
Gum	C03	1	1/0
Mouth	C04, C06	2	2/0
Parotid	C07	1	1/0
Salivary gland	C08	1	1/0
Tonsils	C09	2	1/1
Oropharynx	C10	2	2/0
Esophagus	C15	7	5/2
Stomach	C16	5	5/0
Colon-rectum	C18-20	24	21/3
Colon	C18	14	12/2
Rectum	C20	10	9/1
Gallbladder	C23	1	1/0
Pancreas	C25	12	8/4
Larynx	C32	6	5/1
Trachea, Bronchus and Lung	C33-34	66	52/14
Skin melanoma	C43	19	14/5
Other connective and soft tissue	C49	4	4/0
Breast (female)	C50	29	-/29
Corpus uteri	C54	8	-/8
Ovary	C56	5	-/5
Prostate	C61	36	36/-
Testis	C62	9	9/-
Kidney	C64	15	14/1
Bladder	C67	28	27/1
Brain	C71	6	4/2
Thyroid gland	C73	11	6/5
Multiple myeloma	C90	5	3/2
Lymphoid Leukaemia	C91	4	4/0
Myeloid Leukaemia	C92	4	3/1
**Not otherwise specified**	**C76-80**	**5**	**5/0**

Figure S1. Distribution of de novo malignancies according to period at cancer diagnosis and cancer group

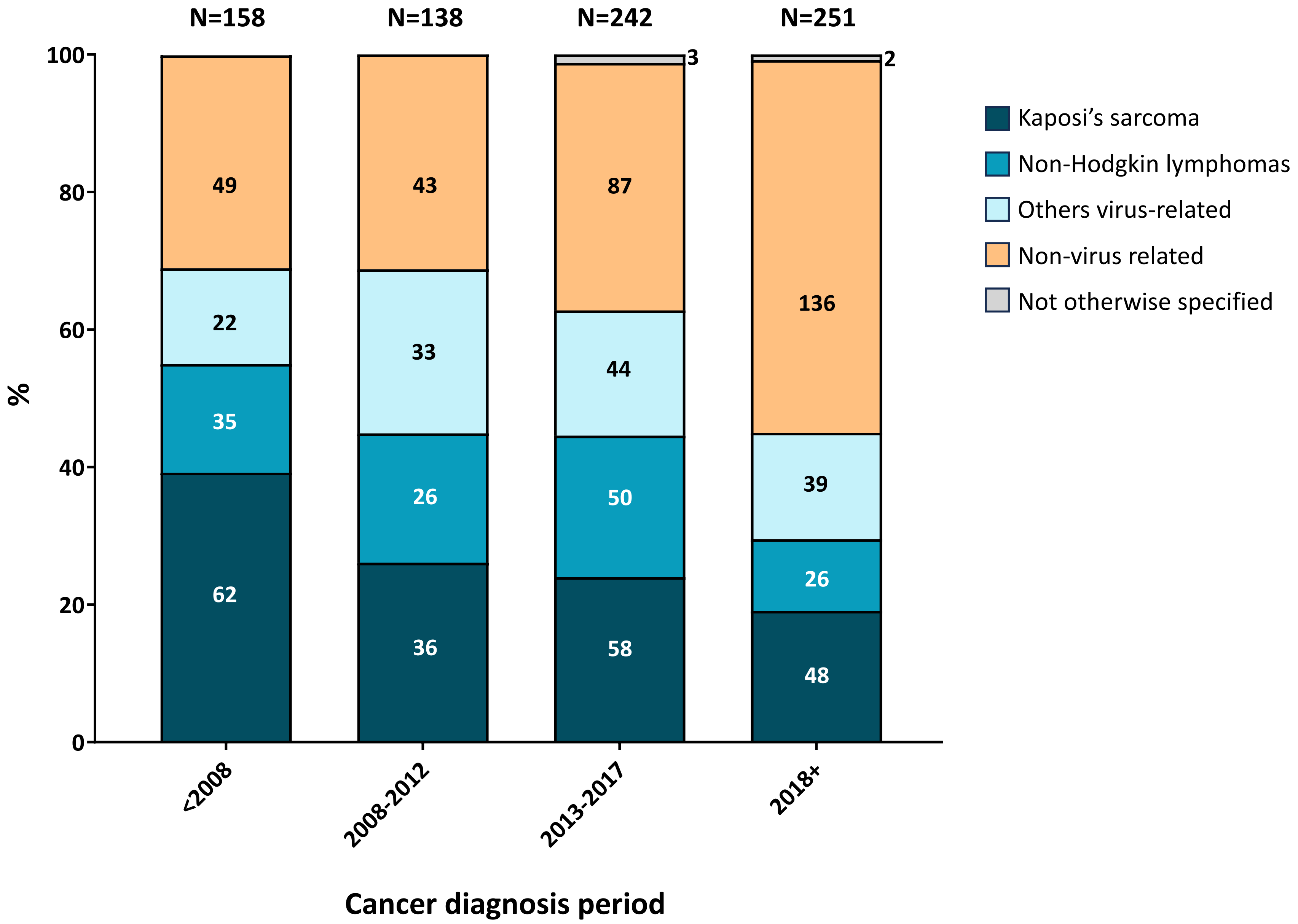

Supplement: Supplementary file 1 — Data S1: Supplementary Information [file IJC-157-1142-s001.pdf]
